# The Botsha Bophelo Adolescent Health Study: A profile of adolescents in Soweto, South Africa

**DOI:** 10.4102/sajhivmed.v18i1.731

**Published:** 2017-09-29

**Authors:** Cari L. Miller, Busisiwe Nkala, Kalysha Closson, Jason Chia, Zishan Cui, Alexis Palmer, Robert Hogg, Angela Kaida, Glenda Gray, Janan Dietrich

**Affiliations:** 1 Faculty of Health Sciences, Simon Fraser University, Canada; 2 Perinatal HIV Research Unit, Soweto, South Africa; 3 Faculty of Health Sciences, University of the Witwatersrand, South Africa; 4 The British Columbia Centre for Excellence in HIV/AIDS, Vancouver, Canada; 5 South African Medical Research Council, Cape Town, South Africa

## Abstract

**Background:**

Youth between the ages of 15 years to 24 years account for almost half of new HIV infections in South Africa.

**Objectives:**

To describe the study details of the Botsha Bophelo Adolescent Health Study (BBAHS) which was an investigation of HIV risk among adolescents living in Soweto, South Africa.

**Methods:**

Eligibility criteria for the BBAHS included being 14 years – 19 years old and living in one of the 41 identified formal and informal areas in the township of Soweto. A cross-sectional survey was developed between investigators and an adolescent community advisory board consisting of previously validated scales and original questions including demographics, sexual and reproductive health, health service utilisation and psychosocial behaviours.

**Results:**

Between 2010 and 2012, interviewers administered surveys among 830 adolescents (57% females), whose median age was 17 years (Q1, Q3: 16, 18), and found that 43% of participants identified their ethnicity as Zulu, 52% reported high food insecurity, 37% reported at least one parent had died, 15% reported living in a shack and 83% identified as heterosexual. Over half of the participants (55%) reported ever having sex (49% of females and 64% of males), 11% of whom initiated sex at < 15 years of age (3% females and 21% males). Almost half (47%) reported ever testing for HIV, 3% (*n* = 12) of whom self-reported being HIV-positive and 33% (*n* = 4) were on antiretroviral therapy.

**Conclusion:**

Our study highlights important individual, relational and structural level determinants of HIV risk for adolescent men and women growing up within HIV hyperendemic settings.

## Introduction

Youth worldwide make up an estimated 45% of all new HIV infections.^1,2^ According to the United Nations, *youth* is defined as persons between 15 years and 24 years of age. Almost two-thirds of the estimated 35.3 million people living with HIV in the world live in sub-Saharan Africa, and approximately 4 million of those are youth (15 years – 24 years).^2^ HIV has particularly affected South Africa, with an estimated 6.3 million people living with HIV. An estimated 2.4 million youth in South Africa have lost one or both of their parents to AIDS.^2^ Furthermore, there is a dramatic rise in HIV infection rates around the age of sexual debut.^3,4,5^

Individual and socio-structural level factors intersect within the lives of South African adolescents^5^ to influence HIV risk, including violence, poverty, substandard housing conditions and food insecurity.^6,7,8,9^ According to the World Health Organization (WHO), *adolescents* are described as persons between 10 years and 19 years of age. Moreover, there is a gendered dimension to HIV vulnerability among youth, with women having three times the HIV infection rates compared to men.^4^ Disproportionate rates of HIV faced by these women are influenced by increased biological susceptibility, gender-based economic inequities, unequal power within relationships and intimate partner and sexual violence.^10,11,12,13^

In South Africa, despite widespread HIV prevention in school and media campaigns, a nation-wide study found that less than half of adolescents knew that condoms were effective in preventing HIV, and overall accurate HIV knowledge was poor.^14^ Adolescent sexual health implies that adolescents possess accurate and relevant knowledge regarding their sexual behaviours and the social and structural factors that influence them.^15,16,17^ Furthermore, for adolescents to achieve positive sexual health, there is a need to scale-up adolescent-friendly sexual and reproductive health (SRH) services and support.^18,19^ There has been a noted gap in available and accessible SRH services that also provide evidence-based HIV prevention tools for adolescents despite this age group’s continued HIV vulnerability.^20,21^ SRH services include, but are not limited to, fertility planning, contraception, unplanned pregnancy care, ante-partum and post-partum care, medical male circumcision, sexual violence prevention and victim support services, as well as sexually transmitted infection (STI) prevention, testing and treatment.^22,23^ In many settings worldwide, youth’s access to HIV and SRH services is poor, partly because of the lack of service delivery through appropriate and acceptable means that reflect the actual risks they face.^15,22,24,25^ Previous research has shown comprehensive sexual health education for youth to be intricately woven with their ability to access user-friendly, high-quality and accurate SRH information and services.^22,25^

Data from a systematic review examining HIV risk factors within 68 epidemiological studies found that up to 16% of youth reported having an STI in the previous 12 months.^3^ Population-based surveys from South Africa indicate that while 4% of men and 16% of women between the ages of 15 years – 24 years are HIV-infected, only 11% and 19%, respectively, within this group were aware of their HIV-positive status.^26,27^ Studies in South Africa found low uptake of HIV counselling and testing (HCT) services despite high awareness and willingness to test for HIV among adolescents.^26,28^

In 2008, responding to the lack of adolescent-friendly HIV and SRH services and the WHO’s call for increased comprehensive HIV prevention strategies for adolescents living in endemic communities,^28^ the Perinatal HIV Research Unit (PHRU) in Soweto, South Africa, opened the Kganya Motsha Adolescent Centre, which loosely translates from Sesotho to English as ‘Shine Young Ones’, solely mandated for the HIV, SRH needs of adolescents (14 years – 19 years).^19^ Simultaneously, major changes in the South African government’s willingness to implement a comprehensive and evidence-based HIV and AIDS prevention strategy were underway because of the mass mobilisation and continued pressure of community advocates and in-country scientists.^11,12^ As part of these efforts, a countrywide HIV testing campaign was launched in 2010.^29^

The Botsha Bophelo Adolescent Health Study (BBAHS) was initiated based on the launch of Kganya Motsha Adolescent Centre and efforts to reduce the impact of HIV among adolescents in Soweto. We hypothesised that adolescents living under increased socio-economic hardship marked by high food insecurity and living in informal housing would be at greater vulnerability to HIV risk and less likely to uptake HCT and other HIV-preventative behaviours. Furthermore, given disproportionate rates of HIV among adolescent women in South Africa, it was hypothesised that gender would play an important role in the uptake of HCT and HIV prevention, with more women accessing these services than men.

Overall, the BBAHS investigated the following four primary objectives:

- to examine individual and socio-structural determinants of HCT
- to evaluate the prevalence and correlates of adolescents’ use of HIV prevention resources, including condom use, medical male circumcision and HCT
- to analyse the relationship between adolescents’ HIV knowledge and their use of HIV prevention resources
- to determine the prevalence, risk and protective factors associated with HIV among adolescents living in Soweto, South Africa.

In this article, we highlight our research approach, describe the data available for analyses and present characteristics of the BBAHS to help address the gaps in HIV and SRH research among adolescent populations and to support other studies within this important demographic.

## Methods

### Study setting

The BBAHS was a cross-sectional, observational study of adolescents (14 years – 19 years) living in the HIV endemic township of Soweto, South Africa. Gauteng census data from 2007 estimated that approximately 7.2% of households in the City of Johannesburg municipality lived in informal settlements or areas.^30^ Soweto is a peri-urban township of the City of Johannesburg, consisting of multiple areas,^31^ where adolescent HIV and SRH services were available through PHRU and the Kganya Motsha Adolescent Centre in Soweto. Soweto, which is located in the south of Johannesburg, has approximately 1.3 million inhabitants, with the majority (98%) being identified as black African. According to 2011 census data, there were 315 116 adolescents aged between 15 years and 19 years living in the City of Johannesburg metropolitan municipality.^31^ Previous work in Soweto had found HIV prevalence of 3.6%, with females having significantly higher rates compared to males (4% vs. 2%, *p* < 0.0001).^19^ In 2010, the BBAHS began data collection. Botsha Bophelo was the Sesotho name given to the study by the adolescent community advisory board (CAB), which is loosely translated to mean ‘Youth and their health’. The CAB intentionally chose a name for the study that did not have HIV in the title because they stated that adolescents in Soweto did not want to be defined by HIV, but rather a positive statement that recalled the right to health of adolescents worldwide.^32^

### Recruitment approach

To test primary hypotheses surrounding gender and socio-economic factors, strategic sampling was used to ensure representation from both formal and informal areas (locally known as informal settlements consisting of housing constructed of metal or other no or low cost materials, and typically having no water or electricity). To achieve this, the research staff who were either current residents of Soweto or were highly familiar with the township helped to collectively identify seven informal areas beyond the 34 enumerated areas within Soweto. A total of 41 areas were identified for the recruitment methodology. Eligibility criteria were as follows: participants should be 14 years – 19 years old and must be living in one of the 41 areas in Soweto.

Research interviewers targeted malls, schools and neighbourhood hang-outs that adolescents frequented in the 41 areas identified in Soweto. Adolescents were given a recruitment card with study contact information including phone numbers through which interviewers could be reached. Because of the nature of our recruitment strategy, we were unable to determine a response or refusal rate. Participants learned of the study through targeted recruitment and from PHRU and Kganya Motsha Adolescent Centre staff and were invited to share the information with their peers. The BBAHS is, therefore, a convenience sample that used targeted recruitment strategies based on geographic location, age and gender characteristics.

Interviews were available by appointment or on a drop-in basis at PHRU and the Kganya Motsha Adolescent Centre. Interview times were also available after school and over the weekends to accommodate adolescent schedules. Some communities were more challenging to recruit from than others, particularly in informal areas where the visibility of adolescents was reduced because of the lack of infrastructure for youth to congregate (i.e. schools, malls, etc.). However, recruiters returned to those areas on multiple occasions to locate and inform adolescents about the project until the targeted number of participants were recruited from each of the 41 areas within Soweto.

### Study procedures

Adolescents who accepted a verbal invitation to participate, which included a brief description of the study procedures, were invited to PHRU or the Kganya Motsha Adolescent Centre for an interview, whichever location was more convenient for the participant. Participants under the age of 18 years were asked to sign an assent form and have a parent or legal guardian sign a consent form for their participation. Adolescents 18 years and above signed independent written informed consent. Following the consent/assent, an interviewer-administered structured survey was conducted with participants. Surveys were conducted electronically, using iPads or desktop computers, and lasted approximately for 45 min to 1 h. iPads provided an alternative to desktop computers that allowed the study to circumvent regular power outages and complete interviews online using 3G network access. Participants were given ZAR 50 reimbursement for transportation and associated participation costs.

### Measures

The survey was developed by the research team and the PHRU adolescent CAB. The adolescent CAB was formed by youth between the ages of 15 years to 24 years, who were connected to youth-focused organisations within Soweto. The role of the adolescent CAB continues to be the evaluation of the PHRU adolescent research studies and to serve as a bridge between the organisation and the wider community of Soweto and as a voice for study participants.^33^

The initial English survey was translated into Zulu, a local language, widely spoken in Soweto, and then back-translated to ensure the accuracy of the translation. Following extensive piloting and training with the multi-lingual research interviewers, challenging words or concepts in English were flagged during the interview process. Many participants chose the English version of the survey and interviewers verbally translated in real time from English to one of the local languages when necessary. For concepts and terminology that remained unclear, two focus group discussions were conducted with adolescents to clarify concepts such as ‘anal sex’ that was more widely understood as ‘chocolate boxing’ within this population. With this new information, local and age-appropriate terminology was inserted for clarity in the survey.

The development of the BBAHS survey involved utilising the experience and resources of previous adolescent research conducted through the PHRU. The BBAHS research team conducted a literature review to determine the most appropriate validated scales and to identify in-country gaps. In addition to SRH questions, previously validated scales were included to measure food insecurity (Cronbach alpha = 0.81),^34,35^ depression scores (CES-D) (Cronbach study alpha = 0.81),^36,37,38^ self-esteem (Cronbach alpha = 0.65),^37,39^ HIV knowledge^40^, trauma (Cronbach alpha = 0.63),^41^ alcohol use^42^ and risk-reduction self-efficacy (study Cronbach = 0.65).^43^ Some questions from the validated scales were modified to better fit the local adolescent population. Sex or gender was assessed by asking participants to identify their sex (male versus female). Socio-demographic information included age, ethnicity and education level. Additional questions within the survey explored sexual beliefs and behaviours, alcohol and drug use, experience of gender-based and other violence, condom and contraceptive use, HCT, male circumcision, HIV and antiretroviral therapy (ART) knowledge, and self-reported STI and HIV status.

Because of the sensitive nature of many of the questions, trained counsellors and social workers were accessible through PHRU or the Kganya Motsha Adolescent Centre if a question triggered an emotional response or if participants needed further support. All participants were informed about Kganya Motsha Adolescent Centre’s adolescent-friendly mandate and were invited for free HCT at the time of recruitment, which likely explains the higher proportion of adolescents who reported testing in our sample than in other adolescent samples in South Africa. The BBAHS received ethical approval from the respective Research Ethics Boards (REBs) at the University of the Witwatersrand in South Africa and Simon Fraser University in Canada.

### Data and statistical analyses

Data were entered directly into Survey Monkey,^44^ a low cost, online survey design and data collection tool. Data were anonymised through the use of numerical IDs. No names or identifiers were placed online into the Survey Monkey system. A study data manager transferred data weekly to a secure, password- and firewall-protected server. Database and all analyses were conducted using SAS 9.4. To compare all characteristics by gender, Chi-square tests or Fisher’s exact tests were conducted for categorical variables, and Wilcoxon’s rank-sum tests were conducted for continuous variables. All statistical tests were considered significant at α < 0.05.

#### Ethical considerations

This study was approved by Research Ethics Boards of the University of British Columbia (Providence Health Care) (H13-01845) and Simon Fraser University in British Columbia, Canada (2016s0048), as well as the University of Witwatersrand in Johannesburg, South Africa (M090449).

## Results

After extensive data cleaning, a final sample of 830 adolescent participants was reached. Table 1 summarises key socio-demographic characteristics overall and by gender. Participants within the survey include more (57%) females than males, with a median age of 17 (Q1, Q3: 16, 18). Most adolescents identified as heterosexual (92%), over half (52%) reported high food insecurity, 15% (*n* = 122) reported living in a shack and 12% (*n* = 101) reported living in Reconstruction and Development Programme (RDP) housing.^45^ (The Reconstruction and Development Programme [RDP] is a policy framework for integrated and coherent socio-economic progress.) More than a third (37%, *n* = 306) reported one or both parental deaths and most reported their ethnic identity as Zulu (43%). Compared to adolescent men, adolescent women were significantly (*p* ≤ 0.05) more likely to report a sexual identity other than straight (*p* = 0.02) and live in a brick house with family compared to other less stable forms of housing (*p* = 0.04).

**TABLE 1:** Socio-demographic characteristics of male and female adolescents (ages 14–19 years) in the Botsha Bophelo Adolescent Health Study (*N* = 830).

Characteristic	Overall			Sex				*p*
				Male (*n* = 355)		Female (*n* = 475)		
	*n*	%	†	*n*	*%*	*n*	*%*	
**Age group (years)**	**-**	**-**	**10**	**-**	**-**	**-**	**-**	**0.2196**
≤ 15	179	21.8	-	86	24.7	93	19.7	-
16–17	233	28.4	-	97	27.9	136	28.8	-
17–19	408	49.8	-	165	47.4	243	51.5	-
**Sexual orientation**	**-**	**-**	**88**	**-**	**-**	**-**	**-**	**0.0199**
Straight	686	92.5	-	302	95.6	384	90.1	-
Gay or Lesbian	21	2.8	-	6	1.9	15	3.5	-
Bisexual	35	4.7	-	8	2.5	27	6.3	-
**Ethnicity**‡	**-**	**-**	**2**	**-**	**-**	**-**	**-**	**-**
Zulu	359	43.4	-	171	48.2	188	39.8	-
Sotho	128	15.5	-	42	11.8	86	18.2	-
Xhosa	116	14.0	-	51	14.4	65	13.7	-
Tswana	86	10.4	-	27	7.6	59	12.5	-
Mixed race	29	3.5	-	16	4.5	13	2.8	-
Tsonga	29	3.5	-	13	3.7	16	3.4	-
Pedi	23	2.8	-	11	3.1	12	2.5	-
Swati	22	2.7	-	10	2.8	12	2.5	-
Venda	20	2.4	-	7	2.0	13	2.8	-
White	8	1.0	-	5	1.4	3	0.6	-
Ndebele	7	0.9	-	2	0.6	5	1.1	-
Other	1	0.1	-	0	0.0	1	0.2	-
**Housing**	**-**	**-**	**0**	**-**	**-**	**-**	**-**	**0.0417**
Brick house owned by family	584	70.4	-	236	66.5	348	73.3	-
RDP House	101	12.2	-	55	15.5	46	9.7	-
Shack	123	14.8	-	52	14.7	71	15.0	-
Other	22	2.7	-	12	3.4	10	2.1	-
**Food insecurity**	**-**	**-**	**3**	**-**	**-**	**-**	**-**	**0.1011**
Low	181	21.9	-	65	18.4	116	24.5	-
Medium	214	25.9	-	94	26.6	120	25.4	-
High	432	52.2	-	195	55.1	237	50.1	-
**Any parents dead**	**-**	**-**	**0**	**-**	**-**	**-**	**-**	**0.7846**
Yes	306	36.9	-	129	36.3	177	37.3	-
No	524	63.1	-	226	63.7	298	62.7	-

RDP, Reconstruction and Development Programme.

† Missing/do not know/prefer not to answer; ‡, Fisher’s exact test conducted; Chi-square test conducted if not labelled.

Table 2 presents descriptive characteristics regarding HIV knowledge, health service and HIV–HCT utilisation. In general, BBAHS participants had moderate HIV-related knowledge, with adolescent women scoring an average of 78% (14/18) and adolescent men scoring 72% (13/18) on a previously validated 18-item HIV knowledge questionnaire.^40^ Only 20% of participants could correctly identify that chimpanzees were the zoonotic reservoirs of HIV-1. Further, 29% believed that HIV was a conspiracy, and over half (51%) were unsure of the origins of HIV.^46^ Males within our study scored significantly lower on the HIV knowledge scale (*p* < 0.001) and were less likely to correctly identify the origins of HIV compared to females (*p* < 0.001).

**TABLE 2:** HIV knowledge, self-esteem, health service and HIV–VCT counselling and testing utilisation among male and female Botsha Bophelo Adolescent Health Study participants (*N* = 830).

Characteristic	Overall (*n* = 830)			Sex				*p*
				Male (*n* = 355)		Female (*n* = 475)		
	*n*	%	†	*n*	%	*n*	%	
**HIV knowledge**	**-**	**-**	**-**	**-**	**-**	**-**	**-**	**-**
HIV knowledge % (HIV-KQ-18) (Med, Q1–Q3)	78	67–83	0	72	61–83	78	67–89	<0.0001§
Self-Esteem Scale (Med, Q1–Q3)¶	3.7	3.4–4.1	2	3.6	3.3–4.0	3.7	3.5–4.1	0.0072§
HIV origin knowledge (Where do you think HIV came from?)	-	-	0	-	-	-	-	<0.0001
Monkeys/chimpanzees	168	20.2		95	26.8	73	15.4	-
Conspiracy theory (e.g. space, the United States government…)	238	28.7		114	32.1	124	26.1	-
Other/unsure	424	51.1		146	41.1	278	58.5	-
Healthcare utilisation	-	-	-	-	-	-	-	-
**Used healthcare service in the past six months (P6M)**	**-**	**-**	**12**	**-**	**-**	**-**	**-**	**0.4957**
No	594	72.6	-	257	73.9	337	71.7	-
Yes	224	27.4	-	91	26.2	133	28.3	-
**Top two reasons for using healthcare service (*n* = 224, multiple choices)**	**-**	**-**	**14**	**-**	**-**	**-**	**-**	**-**
Concerned about HIV	52	24.8	-	25	30.1	27	21.3	0.1740
Flu-like symptoms	25	11.9	-	13	15.7	12	9.5	0.1458
Other	146	69.5	-	54	65.1	92	72.4	0.2560
HIV testing	-	-	-	-	-	-	-	-
**Ever tested for HIV?**	**-**	**-**	**26**	**-**	**-**	**-**	**-**	**0.0901**
No	426	53.0	-	192	56.5	234	50.4	-
Yes	378	47.0	-	148	43.5	230	49.6	-
**HIV test results (*n* = 378)**	**-**	**-**	**15**	**-**	**-**	**-**	**-**	**0.4635**‡
Positive	12	3.3	-	6	4.3	6	2.7	-
Negative	349	96.1	-	135	95.7	214	96.4	-
Unsure	2	0.6	-	0	0.0	2	0.9	-
**On antiretroviral therapy? (*n* = 12)**	**-**	**-**	**1**	**-**	**-**	**-**	**-**	**1.0000**‡
No	7	63.6	-	3	60.0	4	66.7	-
Yes	4	36.4	-	2	40.0	2	33.3	-
**Reason for testing for HIV (*n* = 376)**	**-**	**-**	**2**	**-**	**-**	**-**	**-**	**0.0412**
Wanted to know HIV status	216	57.5	-	72	49.0	144	62.9	-
Had unprotected sex	30	8.0	-	12	8.2	18	7.9	-
Was not feeling well	17	4.5	-	7	4.8	10	4.4	-
Other	113	30.1	-	56	38.1	57	24.9	-
**Reasons for not testing for HIV (*n* = 420)**	**-**	**-**	**0**	**-**	**-**	**-**	**-**	**0.0798**
Fear of being positive	70	16.7	-	22	13.9	48	18.3	-
Fear of dying	47	11.2	-	25	15.8	22	8.4	-
Fear of being rejected	20	4.8	-	9	5.7	11	4.2	-
Other	283	67.4	-	102	64.6	181	69.1	-

VCT, voluntary counseling and testing; HIV-KQ-18, HIV Knowledge Questionnaire; Med, median; P6M, past six months.

† Missing/do not know/prefer not to answer; ‡, Fisher’s exact test conducted; Chi-square test conducted if not labelled; §, Wilcoxon’s rank-sum test conducted; ¶, Higher scores = lower self-esteem.

Previous analyses using the BBAHS survey found no gender differences between males and females with respect to healthcare utilisation (26% vs. 28%; *p* = 0.447).^47^ Of those that sought healthcare services (*n* = 224), 30% of males and 21% of females reported being concerned about HIV as the reason for seeking healthcare.

Almost half [378 (47%)] of the participants reported ever having an HIV test. Among those, 3% (*n* = 12) reported being HIV-positive and two (0.6%) were unsure of their HIV status. Four out of the 12 (33%) adolescents reported being on ART. Of those that tested for HIV, the main reason for both males and females was that they wanted to know their HIV status (57% overall). The main reasons for not being tested included fear of being positive (14% of males and 18% of females) and fear of dying (16% of males and 8% of females).

Adolescents who identified their sexuality as other than heterosexual had significantly higher HIV prevalence (13.8% vs. 2.3%; *p* = 0.002).^48^ Furthermore, 17 (5%) participants reported being unsure of their HIV status, and only one-third of the HIV-positive participants reported accessing ART.

Table 3 provides descriptive statistics on select sexual reproductive health outcomes, beliefs and behaviours. About half (55%) of the participants reported ever having consensual sex, with significantly (*p* < 0.01) more males (64%) than females [233 (49%)] reporting sexual activity. Of those who had ever had sex, more than one-third of adolescents reported being abstinent in the past six months (36%), with no significant difference between males and females (*p* = 0.45). Among adolescents who had sex, 23% (41% of males and 7% of females) reported consensual sexual activity before 15 years (*p* < 0.01), and 55% reported inconsistent condom use with no significant difference by gender (*p* = 0.91). More male adolescents reported having more than two sexual partners in the past six months (*p* < 0.01). Females self-reported higher STI diagnoses compared to males (27% vs. 19%, *p* = 0.50), and 8% (*n* = 37) of females reported ever being pregnant compared to 11% (*n* = 22) males reporting ever making someone pregnant (*p* = 0.13). Of those who answered questions about contraception (*n* = 506), 40% (*n* = 334) reported condom use as their primary STI and pregnancy prevention mechanism and 7% (*n* = 56) reported no family planning method.

**TABLE 3:** Sexual and reproductive health of the male and female Botsha Bophelo Adolescent Health Study participants (*N* = 830).

Characteristic	Overall (*n* = 830)			Sex				*p*
				Male (*n* = 355)		Female (*n* = 475)		
	*n*	%	†	*n*	%	*n*	%	
**Ever had sex**			**0**	**-**	**-**	**-**	**-**	**<0.0001**
No	369	44.5	-	127	35.8	242	50.9	-
Yes	461	55.5	-	228	64.2	233	49.1	-
**Age at first sex (*n* = 461)**	**-**	**-**	**83**	**-**	**-**	**-**	**-**	**<0.0001**
15 or older	289	76.5	-	111	59.4	178	93.2	-
Before the age of 15	89	23.5	-	76	40.6	13	6.8	-
**Inconsistent condom use (*n* = 461)**	**-**	**-**	**0**	**-**	**-**	**-**	**-**	**0.9124**
No	192	44.8	-	95	45.0	97	44.5	-
Yes	237	55.2	-	116	55.0	121	55.5	-
**Self-reported STI (*n* = 461)**	**-**	**-**	**0**	**-**	**-**	**-**	**-**	**0.0473**
No	356	77.2	-	185	81.1	171	73.4	-
Yes	105	22.8	-	43	18.9	62	26.6	-
**Ever been or made someone pregnant? (*n* = 461)**	**-**	**-**	**85**	**-**	**-**	**-**	**-**	**0.0130**
No	317	84.3	-	174	88.8	143	79.4	-
Yes	59	15.7	-	22	11.2	37	20.6	-
**Sex in the P6M (*n* = 461)**	**-**	**-**	**16**	**-**	**-**	**-**	**-**	**0.4471**
No	161	36.2	-	82	38.0	79	34.5	-
Yes	284	63.8	-	134	62.0	150	65.5	-
**More than two sexual partners in P6M (*n* = 284)**	**-**	**-**	**8**	**-**	**-**	**-**	**-**	**<0.0001**
No	178	64.5	-	56	44.1	122	81.9	
Yes	98	35.5	-	71	55.9	27	18.1	
**Ever been circumcised (*n* = 355)**	**-**	**-**	**131**	**-**	**-**	**-**	**-**	**-**
No	-	-	-	132	58.9	-	-	-
Yes	-	-	-	92	41.1	-	-	-
**Birth control methods (multiple choices)**	**-**	**-**	**0**	**-**	**-**	**-**	**-**	**-**
Male condoms	334	40.2	-	191	53.8	143	30.1	<0.0001
No family planning	56	6.8	-	18	5.1	38	8.0	0.0960
Injectable	45	5.4	-	2	0.6	43	9.1	<0.0001
Other (i.e. birth control pill)	71	8.6	-	51	14.4	20	4.2	<0.0001

STI, sexually transmitted infection; P6M, past six months.

† Missing/do not know/prefer not to answer.

Table 4 presents descriptive statistics of substance use, traumatic experiences and depression symptomatology among BBAHS participants. Data show that just over half (64%) of adolescents drank alcohol in the past six months and 21% (*n* = 112) used alcohol more than once per month, 16% (*n* = 134) ever smoked marijuana and 6% (*n* = 53) had ever used illicit drugs other than marijuana. The number of males reporting alcohol and drug use was significantly higher than females (*p* < 0.01). Using the CES-D scale and a cut-off point of 24 or higher, 35% (288) of BBAHS participants met the criteria for depression,^49^ with no significant difference by gender (*p* = 0.16). Adolescent participants reported experiencing an average of seven (out of a possible 19) potentially traumatic experiences, including 13% (*n* = 108) reporting forced sexual intercourse and over half (58%) reporting ever experiencing physical violence.

**TABLE 4:** Alcohol use, drug use, trauma experiences and depression symptomatology among Botsha Bophelo Adolescent Health Study participants (*N* = 830).

Characteristic	Overall (*n* = 830)			Sex				*p*
				Male (*n* = 355)		Female (*n* = 475)		
	*n*	%	†	*n*	%	*n*	%	
**Use of alcohol in the past six months**	**-**	**-**	**11**	**-**	**-**	**-**	**-**	**0.1557**
No	294	35.9		116	33.1	178	37.9	-
Yes	525	64.1		234	66.9	291	62.1	-
**Alcohol frequency in past six months (*n* = 525)**	**-**	**-**	**3**	**-**	**-**	**-**	**-**	**<0.0001**
Less than once per month	410	78.5		163	69.7	247	85.8	-
More than once a month	112	21.5		71	30.3	41	14.2	-
**Ever used drugs (other than marijuana)**	**-**	**-**	**0**		**-**	**-**	**-**	**<0.0001**
No	777	93.6		317	89.3	460	96.8	-
Yes	53	6.4		38	10.7	15	3.2	-
**Ever smoked marijuana**	**-**	**-**	**0**	**-**	**-**	**-**	**-**	**<0.0001**
No	696	83.9		257	72.4	439	92.4	-
Yes	134	16.1		98	27.6	36	7.6	-
**Depression (CES-D, add score)**	**-**	**-**	**12**	**-**	**-**	**-**	**-**	**0.1565**
No	530	64.8		237	67.5	293	62.7	
Yes	288	35.2		114	32.5	174	37.3	
**Ever experienced physical violence?**	**-**	**-**	**0**	**-**	**-**	**-**	**-**	**0.1876**
No	349	42.1		140	39.4	209	44.0	-
Yes	481	57.9		215	60.6	266	56.0	-
**Ever had someone force you to have sexual intercourse?**	**-**	**-**	**8**	**-**	**-**	**-**	**-**	**0.4053**
No	714	86.9		308	88.0	406	86.0	-
Yes	108	13.1		42	12.0	66	14.0	-
**Number of potentially traumatic events experienced (Med, Q1–Q3)**	**7**	**5–9**	**63**	**7**	**5–9**	**7**	**5–9**	**0.1844**‡

CES-D, Center for Epidemiological Studies Depression; Med, median.

† Missing/do not know/prefer not to answer; ‡, Wilcoxon’s rank-sum test conducted.

## Discussion

The BBAHS has contributed new knowledge regarding HIV prevalence among sexual minority adolescents in South Africa^48^ and the potential use of social media and cell-based technologies for HIV prevention within adolescent populations.^50^ The study contributes nuanced information to the complex psychosocial factors affecting HIV risk and the sexual health of adolescents in South Africa. Moreover, the BBAHS helped address the research gap in prevention science within adolescent populations and will support best practices for HIV prevention among youth globally.

Community-level sampling within informal areas offers an opportunity to better understand adolescent populations living in informal areas of Soweto. A significant proportion of participants reported living in a shack, almost half met the criteria for being food insecure and one-third had experienced the loss of one or both parents. While the BBAHS does not claim to be representative of all adolescents living in Soweto, these data underscore the fact that adolescents coming of age in this community face many socio-economic challenges that are occurring alongside endemic levels of HIV.

Similar to other studies in South Africa, among the BBAHS participants, more males than females reported sexual activity and more than one-third reported sexual activity in the past six months.^51,52,53^ A small proportion of participants reported having more than one sexual partner in the past six months, with twice as many males as females, and a significantly higher number of adolescent males reported initiating sex at age 15 years and younger. Importantly, more than half of the sexually active participants reported inconsistent condom use, a concerning finding given the high HIV risk in this demographic and underscoring the need to implement additional and accessible HIV prevention technology tools within this population.

A national HIV study in South Africa found that only about 24% of youth had accurate knowledge about HIV transmission and prevention.^14^ In the BBAHS, male and female participants scored 72% and 78%, respectively, on HIV knowledge, suggesting a higher level of HIV knowledge among BBAHS participants than in the national sample. However, caution regarding this interpretation is urged because little is known about the type and level of knowledge necessary to sustain prevention goals within this demographic. Despite relatively high HIV knowledge scores among BBAHS participants, only 20% could correctly link chimpanzees to the origins of HIV and almost one-third believed in a conspiracy theory as the source of the HIV pandemic.^46^ This analysis regarding correctly identifying the origins of HIV further indicated that older adolescents and females were more likely to correctly identify the origins of HIV. This is significant as HIV origin conspiracies have previously been linked to low HIV testing and increased unprotected sex,^54,55^ warranting increased access to accurate scientific knowledge about HIV origins.^56^ These data indicate a gap in HIV knowledge among adolescents in South Africa and the need to further explore the types, levels of knowledge necessary and innovative methods of delivery of HIV information to reverse the impact of the epidemic on this demographic. It may be the case that the scale-up of HIV education programming through multiple sources (e.g. television, billboards and radio) is perhaps not reaching younger adolescents and adolescent men to the same extent as those in older age categories.^14^ Alternatively, adolescents may be experiencing information fatigue around biomedical constructs of HIV or that an increase in HIV knowledge is affected by pervasive sexual violence against women.

HCT among BBAHS participants was much higher compared to national estimates.^57^ This is likely because adolescents were recruited at PHRU and the Kganya Motsha Adolescent Centre and were offered free HCT. These data suggest that engaging adolescents in HIV research can have positive public health benefits when combined with evidence-based interventions in addition to collecting data. Moreover, a surprising proportion of adolescents were willing to test when offered the opportunity to do so in a safe and adolescent-friendly environment. However, more than half of the adolescents had never had an HIV test and many expressed fears surrounding learning their status. It is imperative that public health systems should do more to remove the stigma of HIV testing and break down fears and misconceptions regarding knowing one’s HIV status. Having access to adolescent-friendly HCT sites may be particularly important given the 2012 study indicating that although 90% of South African adolescents and youth could identify a location they could get tested for HIV, only 59% of those had ever been tested.^14^ Our findings among HIV-infected study participants underscore the need to do more to connect adolescents to HIV prevention and treatment services, particularly in light of the evidence for treatment as prevention.^18,58,59,60^ Moreover, these data highlight a need to connect sexual minority adolescents to preventative health services.

The majority of adolescents in our study used alcohol in the past six months, adolescent men were significantly more likely to take alcohol more than once per month and use marijuana and other illicit drugs compared to adolescent women. Previous research indicates that reduced alcohol use among men is associated with decreases in the perpetuation of intimate partner violence and increased partner communication and equality in sexual decision-making in relationships.^61^ Interventions addressing problematic alcohol use among university students in South Africa have been successful in reducing symptoms of depression.^62^ Similar to other studies among South African adolescents, we found that about one-third of BBAHS participants experienced high depression symptomatology (measured through the CES-D scale).^63^ This indicates an important point of intervention for adolescents who may be initiating sex and substance use in an HIV hyperendemic community where high levels of depression have been associated with problematic alcohol use and risky sexual practices such as inconsistent condom use.^64^

Adolescents in our study experienced a high number of potentially traumatic events, including high levels of physical violence. These findings highlight the complexities of adolescent lives within the context of high HIV risk, indicating a need for further investigation and investments in comprehensive multi-level adolescent-centred interventions that will address co-factors including alcohol and drug use,^7,61^ mental illness and violence^11,12,13,65^ experienced by adolescents living in the peri-urban setting of Soweto, South Africa.

### Limitations of the study

This study utilised a non-random sample, which limits the ability to infer findings to the general population. The modest reimbursement offered for study participation may have yielded an overrepresentation of individuals in need of financial assistance. This study relied on self-reported data; therefore, socially desirable responding and recall bias may have affected some of the study’s variables. The cross-sectional nature of this study infers that causal relationships cannot be determined.

## Conclusion

Despite persistently high levels of HIV prevalence within South Africa and increased attention to the gendered epidemic, gaps in addressing the specific needs of adolescents remain.^2,23,66^ Our results provide efforts that holistically address the HIV epidemic through individual, community and structural levels.
